# *Antrodia cinnamomea* is a potentially effective complementary medicine for adjuvant therapy against breast cancer with bone metastasis: A case report

**DOI:** 10.1097/MD.0000000000020808

**Published:** 2020-07-02

**Authors:** Huei Long, Chi-Tan Hu, Vesna Prijatelj, Ching-Feng Weng

**Affiliations:** a Department of Life Science and Institute of Biotechnology, National Dong Hwa University; b Research Centre for Hepatology, Hualien Tzu Chi Hospital, Buddhist Tzu Chi Medical Foundation; c School of Medicine, Tzu Chi University, Hualien, Taiwan; d University Medical Centre, Informatics Department, Ljubljana, Slovenia; e Department of Basic Medical Science, the Center of Translational Medicine; f Department of Basic Medical Science, Institute of Respiratory Disease, Xiamen Medical College, Xiamen, Fujian, China.

**Keywords:** alkaline phosphatase, *Antrodia cinnamomea*, bone metastasis, breast cancer, carcinoma antigen 15-3, complementary medicine, lactate dehydrogenase

## Abstract

**Rationale:**

Palbociclib (PAL) is a first-in-class selective inhibitor of the cyclin-dependent kinases 4 (CDK4) and CDK6 and is indicated for the treatment of hormone receptor (HR)-positive/human epidermal growth factor receptor 2 (HER2)-negative metastatic breast cancer (MBC) in combination with fulvestrant (FUL) in postmenopausal women. *Antrodia cinnamomea* (AC), a well-known Chinese folk medicine in Taiwan, possesses numerous biological capabilities, most notably an anti-tumor effect. However, the clinical use of AC as complementary medicine combined with adjuvant therapy is unexplored. In this case report, we evaluated AC combined with PAL plus FUL to reduce the tumor burden in an MBC patient.

**Patient concerns:**

A Slovenian woman diagnosed with relapsed bone metastases of breast cancer (BC) was unable to undergo surgery and refused radiation therapy due to fear of side effects; she also feared the side effects of adjuvants. However, she was eager to live with a high quality of life.

**Diagnosis:**

Stage IV, HR-positive/HER2-negative BC with relapse of bone metastases.

**Interventions:**

After diagnosis of relapse of bone metastases, she received adjuvant with PAL plus FUL. Additionally, she chose to take AC orally (10 g/d).

**Outcomes:**

The pain was mostly relieved, and the side effects of adjuvant therapy reduced. Magnetic resonance imaging revealed reduction of tumor size at the fifth month of adjuvant therapy plus AC. After 14 months of adjuvant therapy plus AC, the tumors at the thoracic vertebrae T1 and T3 were found to have shrunk from 35.2 and 12.0 mm to 28.1 and 9.9 mm, respectively. Remarkably, no further metastases were observed.

**Lessons:**

According to the circulating tumor cells (CTCs) test data, AC had better anti-tumor efficacy on active tumor cells than PAL plus FUL. Thus, AC could be an effective complementary medicine for adjuvant therapy in patients with HR-positive/HER2-negative MBC. Interestingly, continued elevation of carcinoma antigen 15-3 and lactate dehydrogenase levels but decreasing levels of alkaline phosphatase were observed, which may be indicative of the potent efficacy of treatment resulting in massive tumor cell death. The CTCs test may be a sensitive approach to monitor the progression of BC and subsequently evaluate the efficiency of therapy.

## 1 Introduction

Breast cancer (BC) is the most commonly diagnosed cancer and the leading cause of cancer death among women. There were about 2.1 million newly diagnosed cases of BC in women in 2018, accounting for almost 1 in 4 cancer cases among women worldwide.^[1]^ Bone is the most common site of metastasis in patients with BC, with up to 75% of patients with stage IV BC developing skeletal metastases.^[2]^ The median overall survival (OS) in patients with BC and bone metastasis based on axillary diagnosis is 5.62 years.^[3]^ Clinically, endocrine therapy is the preferred first-line treatment option for hormone receptor (HR)-positive/human epidermal growth factor receptor 2 (HER2)-negative metastatic breast cancer (MBC).^[4]^ Palbociclib (PAL), an oral inhibitor of cyclin-dependent kinases (CDK4/6), is a first-in-class drug indicated for the treatment of HR-positive/HER2-negative advanced or MBC in combination with fulvestrant (FUL) in postmenopausal women, as the combination of PAL with FUL resulted in superior outcome than FUL alone.^[5,6]^ A pre-specified analysis of OS with PAL and FUL in advanced BC concluded that treatment with PAL-FUL resulted in higher OS than treatment with placebo-FUL. However, the differences in OS among all trial registers were not significant.^[7]^

For management of patients with BC, a sensitive and specific biomarker is helpful for early detection of the disease or for determining the tumor burden. Using routine blood analysis, involving, for example, serum lactate dehydrogenase (LDH) level measurements, as it is the closest prognostic predictive biomarker, could result in a specific strategy for predicting BC without increasing the cost of the diagnostic plan. The increased LDH level is caused by the requirement for metabolic and anaerobic glycolysis in the malignant cells or destruction of tissues or tumor cells, and differences in LDH levels can be used to distinguish between benign and malignant neoplasms of the breast when compared with each other as well as healthy controls.^[8]^ Elevated pre-treatment levels of both alkaline phosphatase (ALP) and LDH are significantly associated with decreased disease-free survival and OS in triple negative BC.^[9]^ A retrospective study has shown that high pre-treatment LDH or both ALP and LDH levels or stably high LDH levels during first line treatment are negative and independent prognostic factors in patients with MBC.^[10]^ Carcinoma antigen (CA15-3) is a better predictive biomarker of BC recurrence than ALP, but the use of both biomarkers together can provide an even better early indicator of recurrence.^[11]^ Moreover, a clinical study suggested that circulating tumor cells (CTCs) are a clinically valuable and independent prognostic marker in newly-diagnosed patients with MBC and can be studied as a unique model to develop tailored treatments for MBC patients.^[12]^

*Antrodia cinnamomea* (AC) is a medicinal fungus which Taiwanese aborigines used widely for treating liver diseases and for protecting liver from food and drug intoxication. It is also a well-known Chinese folk medicine in Taiwan for protection against diverse health-related conditions. AC possesses numerous medicinal properties, especially anti-tumor effects. Cumulative in vitro and in vivo studies of biological activities of AC, such as promotion of apoptosis or autophagy and cell cycle arrest, anti-migration, anti-angiogenesis, and anti-inflammation, have demonstrated that its anticancer effects result from the synergy of miscellaneous bioactive compounds, with each compound being responsible for its specific target or for modulating specific signaling pathways.^[13]^ However, clinical trials of AC as well as investigations into the role of the complementary medicine in the enhancement of PAL plus FUL therapy are unexplored. In this case report, we demonstrated that AC enhanced the therapeutic effects of PAL plus FUL and improved clinical outcomes in a patient with HR-positive/HER2-negative MBC. In addition, alterations in the levels of the cancer related biomarkers CA15-3, LDH, and ALP, were simultaneously observed during the treatment period.

## 2 Case report

The patient gave her informed consent for inclusion before participating in this study. The study was conducted in accordance with the Declaration of Helsinki and approved by the Research Ethics Committee of Hualien Tzu Chi Hospital, Buddhist Tzu Chi Medical Foundation (Case report approval code: CR108-06).

In April 2012, a 50-year old woman underwent a regular mammography, which revealed a tumor (24 x 23 x 21 mm in size) in the upper outer quadrant of her right breast. A surgical biopsy revealed multifocal invasive ductal carcinoma. The tumor was staged as T0N0M0 (stage II, ER 100%, PR 90% positive, HER2 negative). All blood and tumor marker (carcinoembryonic antigen [CEA] and carcinoma antigen 15-3 [CA15-3]) test results were within normal range. In May 2012, a lumpectomy was performed to ensure that there was no lymph node metastasis. She initially received 22 x 2 gray (Gy) radiation as well as 5 x 2 Gy in September 2012. She refused adjuvant therapy with tamoxifen due to a history of endometriosis and was only followed up regularly based on mammography and laboratory and tumor markers test results. There were no signs of disease progression based on these follow-ups.

In September 2016, she underwent computed tomography (CT) and magnetic resonance imaging (MRI) of the spine because of intense pain in left arm. The MRI examination revealed metastasis at C7-T1. However, no metastases to other organs or bones was observed. Furthermore, blood test results and CA15-3 level were still within normal range. In October 2016, resection of the involved C7-T1 and placement of a titanium implant (C6-T2) was performed. She refused adjuvant therapy again, as the guidelines for the diagnosis and treatment of BC did not satisfy the patient. Therefore, she decided to seek alternative approaches for diagnostic and treatment. For 6 months, from October 2016 to March 2017, she took 4 g of dish-cultured AC (Longs Biotech Co. Ltd., Hualien, Taiwan) once daily for her health-related conditions. Patient status was regularly followed up based on CT and MRI in May and June, 2017. In July 2017, CA15-3 level was still within normal range. However, tumor marker CEA level increased (4.6 μg/L) beyond the upper limit (4.0 μg/L).

In February 2018, she underwent cervical spine MRI again because of prominent pain in the right arm due to spinal cord compression. The MRI report revealed bone metastases involving a tumor (35.2 mm in the largest dimension) on the left side vertebral C7-T2 transverse process and lamina and another tumor (12.0 mm in the largest dimension) on the right side of the T3 vertebral body and lamina. The patient refused radiation therapy because of fear of side effects. In March 2018, adjuvant therapy with 500 mg of FUL and 125 mg of PAL was initiated. She was followed up by CT and MRI in April 2018. No further progression was observed.

She had prominent side effects in the first week after FUL administration, including back pain, joint and muscle pain, pain in the legs, a burning sensation at the surgical site on her neck, a burning sensation in the lower stomach, flushing, and weakness. Hand tremors in the morning made her very depressed. She also had conspicuous side effects after PAL administration, such as anemia, neuropathy, bleeding gums, hair thinning or loss, shortness of breath, and sometimes an increased heart rate. Increased levels of the hepatic enzyme alanine transaminase (ALT) were observed due to hepatic toxicity caused by FUL administration. Blood and lymphatic system dysfunctions, such as thrombocytopenia, neutropenia, leukopenia and anemia, and immune system dysfunctions, such as hypersensitivity reactions, caused by FUL and PAL administration were also observed.

**Figure 1:**
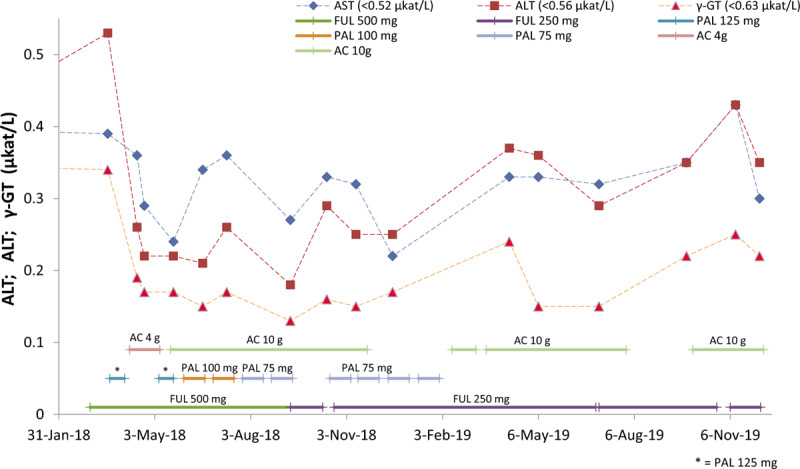
The evaluation of liver function of the breast cancer (BC) patient during the adjuvant and *Antrodia cinnamomea* (AC) therapy period. The liver function of the BC patient during this period was normal, as indicated by the levels of alanine transaminase (ALT), aspartate aminotransferase (AST), and gamma-glutamyltransferase (γ-GT) which were within normal ranges. AC protected the liver against the hepatic toxicity of the adjuvant therapy. AC = *Antrodia cinnamomea,* ALT = alanine transaminase, AST = aspartate aminotransferase, BC = breast cancer, FUL = Fulvestrant, PAL = Palbociclib, γ-GT = gamma-glutamyltransferase.

**Figure 2:**
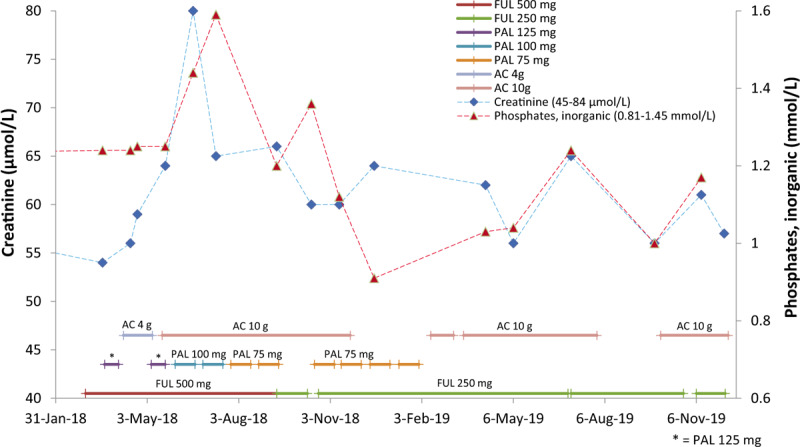
Alterations in serum creatinine and inorganic phosphate levels during the adjuvant therapy plus *Antrodia cinnamomea* (AC). The renal function of the breast cancer patient during the period of adjuvant therapy plus AC was normal, as indicated by the levels of serum creatinine and inorganic phosphate, which were within normal ranges. AC =  *Antrodia cinnamomea,* FUL = fulvestrant, PAL = palbociclib.

**Figure 3:**
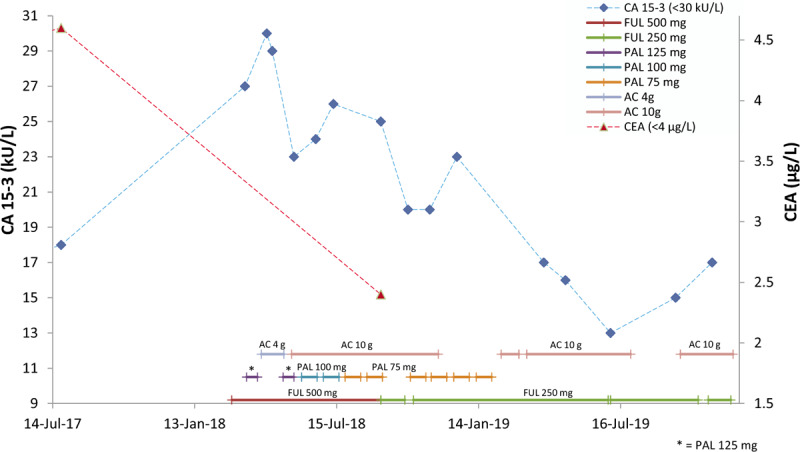
Alterations in cancer biomarker levels during the period of adjuvant therapy plus *Antrodia cinnamomea* (AC). The levels of carcinoembryonic antigen (CEA) and carcinoma antigen 15-3 (CA15-3) fell within normal ranges and showed decline in trend during the period of adjuvant therapy plus AC. Unusually small band elevation of CA15-3 but descending of alkaline phosphatase (ALP) may indicate the potent efficacy of the treatment resulting in continuous and massive tumor cell death. AC = *Antrodia cinnamomea,* CA15-3 = carcinoma antigen 15-3, CEA = carcinoembryonic antigen, FUL = Fulvestrant, PAL = Palbociclib.

**Figure 4:**
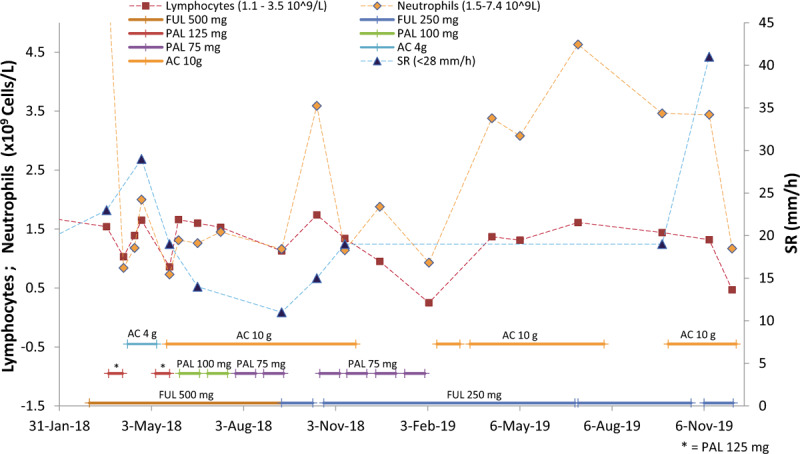
Hematological findings during the period of adjuvant therapy plus *Antrodia cinnamomea* (AC). AC alleviates myelosuppression of adjuvant therapy at Palbociclib (PAL) doses less than 100 mg. The erythrocyte sedimentation rate (SR) fell into within normal range when patient received adjuvant therapy plus 10 g AC per day. AC = *Antrodia cinnamomea,* FUL = fulvestrant, PAL = palbociclib, SR = sedimentation rate.

Due to the side effects of PAL plus FUL, the patient attempted to seek a better clinical outcome, such as an improvement in the quality of life, and safety benefits. In April 2018, she started taking 4 g of AC daily, and her liver functions improved (Fig. 1). To seek better clinical outcomes, such as improvements of OS and the anti-cancer effect, from May to November, 2018, she proceeded to take 10 g of AC daily. Renal function is normally indicated by serum creatinine levels, which remained within normal ranges (Fig. 2) as did her liver functions (Fig. 1). CA15-3 and CEA were reduced during the period of treatment (Fig. 3). Lymphocyte and neutrophil counts returned to normal levels during the periods when the administration of PAL was paused or the dose was reduced (Fig. 4). In June 2018, the efficiency of therapy was evaluated based on the alpha-N-acetylgalactosaminidase enzyme (nagalase) test in a Belgium laboratory. The results showed no cancer cell activity.

In August 2018, follow-up MRI of the cervical spine showed that the tumors had shrunk. In September 2018, the dosages of adjuvant therapy were reduced (FUL from 500 to 250 mg and PAL from 125 to 75 mg) because of the side effects. The grade of those adverse effects due to myelo-suppression decreased as the dose reduced, but they still did not fall within normal range.

**Figure 5:**
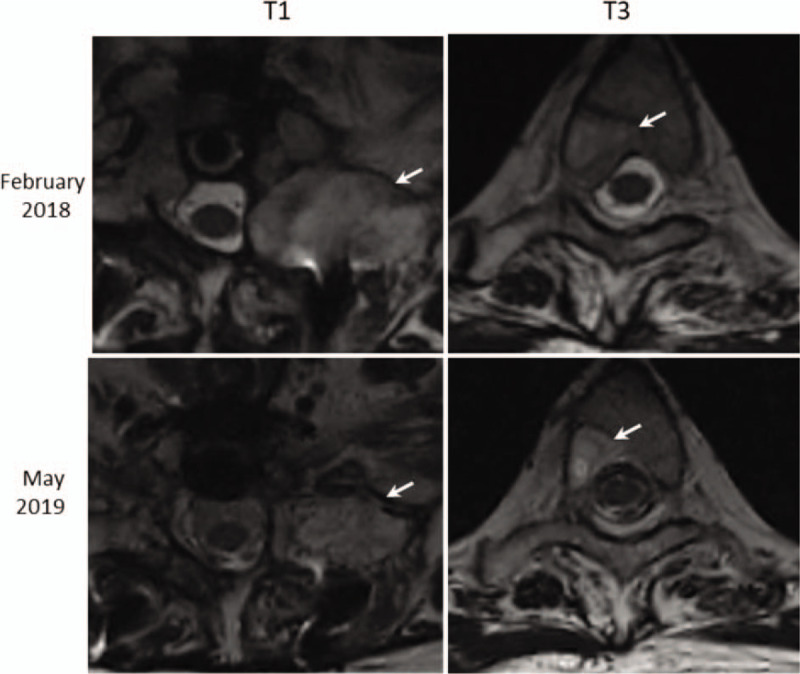
Magnetic resonance imaging of the breast cancer patient with relapse of bone metastases in February, 2018 and on follow-up in May 2019. The tumor on the left side of the vertebral T1 has shrunk from 35.2 to 28.1 mm. Another one on the right side of the T3 vertebrae has shrunk from 12.0 to 9.9 mm. The arrow indicates the tumor.

In November 2018, CT of the chest and abdomen did not show any tumors. In comparison to the previous findings on MRI examination in February 2018, the MRI examination in May 2019 revealed that metastases on the left side of C7-T2 were reduced. Only a shrunken tumor on the left side of T1, which was reduced in the largest dimension from 35.2 to 28.1 mm, remained. The tumor on the right side of the T3 vertebral body had also shrunk from 12.0 to 9.9 mm (Fig. 5). In addition, a bone scan in June 2019 did not reveal any bone metastases in the entire body.

In follow up examination, we conducted more tests to evaluate the efficacy of combined therapy (adjuvant and AC). From October to November, 2018, CTCs test revealed that the number of potential tumor cells in circulation decreased nearly 4-fold (from 20 to 5.75 million) with endocrine therapy (FUL 250 mg with 75 mg PAL) plus 10 g AC per day. In-vitro-vitality reduction in relation to concentration and time with eutherapeutic concentrations of 75 mg PAL and 10 g AC was 15% and 75%, respectively. The ideal outcome is a reduction by 100% in short-term cell culture.

**Figure 6:**
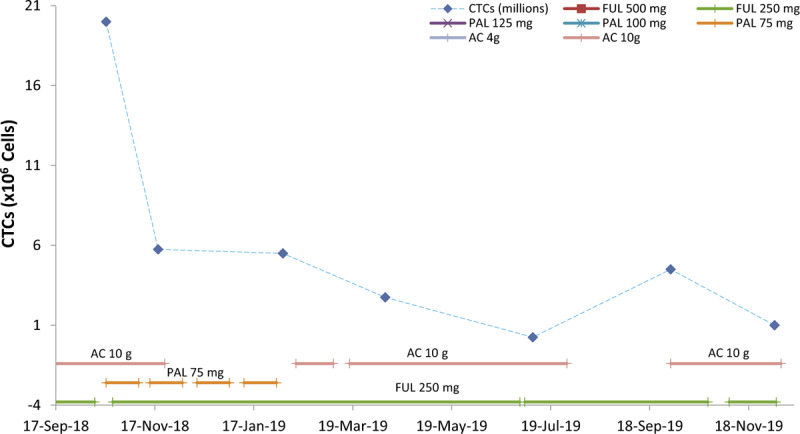
Circulating tumor cells (CTCs) analysis revealed the influence of different combinations of fulvestrant (FUL) (250 mg), palbociclib (PAL) (75 mg), and *Antrodia cinnamomea* (AC) (10 g per day) on breast cancer. The number of CTCs dropped significantly when the patient received adjuvant therapy plus AC 10 g per day. After stopping AC in July, 2019 there was a significant increase in CTC number. Over a period of 2 months, after restarting AC administration and ongoing FUL therapy, we again observed reduced CTC levels. AC = *Antrodia cinnamomea,* CTCs = circulating tumor cells, FUL = Fulvestrant, PAL = Palbociclib.

Therefore, we proceeded to check the influence of 75 mg PAL and 10 g AC in vivo. From November 2018 to January 2019, the patient was administered only 250 mg FUL plus 75 mg PAL without AC. In February 2019, CTCs test revealed that the number of potential tumor cells in circulation dropped from 5.75 to 5.5 million. In comparison to the previous findings in November 2018, the number of potential tumor cells had changed only marginally. From February 2019, the patient ceased use of PAL and was administered only 250 mg FUL plus 10 g AC again. In April 2019, CTCs test revealed that the number of potential tumor cells in circulation dropped from 5.5 to 2.75 million. The number of potential tumor cells had decreased 2-fold in comparison to the previous findings in February 2019. Significantly, the data clearly indicated that AC had an incredible influence on cancer cells in only 2 months. For the next 3 months, the patient proceeded with administration of 250 mg FUL plus 10 g AC per day. In July 2019, the number of potential tumor cells in circulation continually dropped from 2.75 to 0.25 million. Compared to the previous findings in April 2019, the number of potential tumor cells had decreased 11-fold (Fig. 6).

In August 2019, the patient stopped taking AC and was administered only 250 mg FUL. CTCs test in October 2019 revealed that the number of potential tumor cells in circulation had arisen 18-fold (from 0.25 to 4.5 million). She immediately began to take AC again long with 250 mg FUL. In December 2019, the number of potential tumor cells in circulation dropped again from 4.5 to 1 million. The number of potential tumor cells had decreased by more than 4-fold compared with the previous findings in October 2019. This showed that AC may be more efficacious than adjuvant therapy of PAL plus FUL on potential tumor cells.

## 3 Discussion

The patient in this case experienced pain due to 2 reasons. Severe pain was due to spinal cord compression resulting from 2 recurrences of bone metastases. The pain caused by incurable bone metastases negatively impacted mobility, ability to carry out daily tasks, quality of life, and patient mental state. Unexpectedly, upon the second relapse, pain was relieved due to tumor regression after the patient started receiving adjuvant therapy plus AC. According to the follow-up MRI, CT, and whole-body bone scan examination, the tumor had regressed, and no further metastases were found after the initial diagnosis of bone metastases relapse. Tumor shrinking might be uncommon for patients receiving PAL plus FUL. However, in this case, the patient's tumor mass had shrunk visibly during the adjuvant therapy plus AC (Fig. 5) period. According to the results of CTCs analysis, PAL-FUL combined with AC had the most prominent and potent influence on BC cells. In comparison with the influence of PAL or FUL alone, the number of tumor cells in circulation was significantly reduced by administration of either of the 2 drugs (PAL and FUL) or the single drug (FUL) combined with AC (Fig. 6). The implication is that, although the patient was taking a combination of therapies, in case of adverse effects of PAL, FUL plus AC is good alternative approach.

Since malignant cells release enzymatically active nagalase which causes a decrease of GC protein-derived macrophage-activating factor levels to promote immunodeficiency in cancer patient, nagalase is a good marker for monitoring the efficacy of cancer therapy.^[14]^ In this case, nagalase test results showed no activities of cancer cells at the third month of adjuvant therapy plus AC. In the meantime, CA15-3 and CEA levels decreased to normal ranges (Fig. 3). Previously, a double-blind clinical trial evaluated the effects of AC in combination with chemotherapy and showed that the mean 6-month survival rate was not significantly different between the AC and placebo groups due to the short treatment of only 30 days.^[15]^ In this case, the result of the nagalase test and the follow-up MRI examination at the fifth month of adjuvant therapy plus AC revealed that administration of 10 g of AC for longer periods (over 3 to 5 months) was more beneficial for the patient in terms of tumor regression and clinical outcomes.

According to the published information of Faslodex, the adverse reaction frequency in terms of elevated hepatic enzyme (ALT, aspartate aminotransferase (AST), and ALP) levels is very common (≥1/10). In the multicenter, randomized, placebo-controlled, phase III study PALOMA-3 study, ALT increase of any grade was reported in 5.8% (20/345) patients receiving FUL in combination with PAL and in 3.5% (6/172) patients receiving FUL plus placebo. In our case, ALT elevations nearing the upper normal limit were observed at the eighteenth day of administration of 500 mg of FUL. After the patient started taking AC, the levels of ALT, AST, and γ-GT reduced significantly and remained in normal ranges (Fig. 1). This indicated that AC could protect against drug-induced liver injury caused by the hepatotoxicity of FUL plus PAL therapy. In addition, adverse reactions, e.g*.,* infections, fatigue, nausea, stomatitis, diarrhea, vomiting, alopecia, rash, decreased appetite, and pyrexia^[16]^ also improved after the patient started taking AC. However, hematologic adverse modalities, such as neutropenia, leukopenia, anemia, and thrombocytopenia, were not significantly improved when the patient received a dose of 500 mg FUL plus 125 mg PAL. The myelo-suppressive effects were clearly reversed when PAL or FUL administration was paused or the dose reduced. Interestingly, the lymphocyte and neutrophil counts declined due to the cessation of AC administration from November 2018 to February 2019, even at reduced dose of FUL (250 mg) alone or with PAL (75 mg) (Fig. 4).

**Figure 7:**
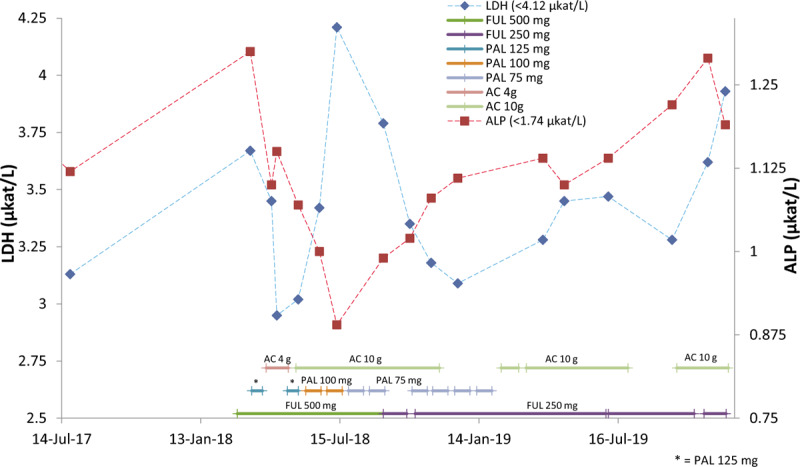
Alterations in serum alkaline phosphatase (ALP) and lactate dehydrogenase (LDH) levels during the period of adjuvant therapy plus *Antrodia cinnamomea* (AC). Unusual continued elevation of LDH levels but descending ALP levels may indicate the potent efficacy of treatment resulting in massive, continuous tumor cell death. AC = *Antrodia cinnamomea,* ALP = alkaline phosphatase, FUL = fulvestrant, LDH = lactate dehydrogenase, PAL = palbociclib.

In general, persistent rise in serum LDH and ALP levels is found because of poor response to treatment, metastasis, or recurrence.^[17]^ The skeletal isoenzyme originates in osteoblasts that release large amounts of the enzyme when bone repair activity occurs, for example, with bone metastases.^[11]^ A clinical study has suggested using ALP as a biomarker to predict patients with or without relapse in liver and bone metastases.^[18]^ In our case, persistent elevation in serum LDH and ALP levels was observed due to relapse of bone metastases in February 2018 (Fig. 7). Firstly, both serum LDH and ALP levels dropped very significantly after the patient started receiving adjuvant therapy plus 4 g AC per day. However, the level of ALP continued to decrease, but, unusually, the level of LDH started rising when the patient further received adjuvant therapy plus 10 g AC per day. According to the result of the nagalase test at the second month of adjuvant therapy plus 10 g AC, no cancer cell activity was observed, indicating that the rising level of LDH may correlate with massive destruction of tumor cells, not with further cancer progression. In the meantime, the level of ALP continued to decrease, indicating that progression of bone metastases may gradually stop due to destruction of tumor cells. This conjecture also was confirmed by the MRI imaging of tumor reduction in size in August 2018, at the fifth month of adjuvant therapy plus 10 g AC per day. A similar situation of unusually increasing LDH levels was observed in a clinical study by Agrawal et al in 2016. The elevation in serum LDH levels was found in the first week following surgery due to radical destruction of cancer cells resulting in the release of enzyme into the circulation.^[17]^ The continued descending level of ALP and the continued increasing level of LDH reversed their trends after reduction of PAL dose from 100 to 75 mg. The elevated level of serum ALP and continued descending level of LDH may imply that bone growth and destruction of tumor cells became more moderate. Herein, ALP and LDH, when measured together, may also be useful and powerful in monitoring the therapeutic efficacy in patients with BC and bone metastases, especially when therapy exerts potent therapeutic efficacy resulting in unusual elevation of LDH levels.

CA15-3 is a tumor marker used to monitor the therapeutic efficacy in patients with BC. In our case, the persistent elevation of serum CA15-3 level was also observed due to relapse of bone metastases, although the alteration was still in normal range (Fig. 3). In contrast, CEA levels increased up to 4.6 μg/L and rose above the upper limit (4.0 μg/L) before relapse of bone metastases was confirmed using MRI in February 2018 (Fig. 3), which is more sensitive marker than CA15-3. In the continuous decline of CA15-3, the small band rising of CA15-3 occurred when the patient received adjuvant therapy plus 10 g of AC daily. This indicated that massive tissue or cell death or cell damage had occurred. MRI of the cervical spine at the fifth month of adjuvant therapy plus 10 g AC showed that the tumors had reduced in size. This might support that the small band rising of CA15-3 was caused by massive BC cell death. Another evidence supporting this conjecture is the dramatically declining number of CTCs as measured after second adjuvant therapy plus 10 g AC (from October to November, 2018) (Fig. 6).

Why did the small band rising of CA15-3 level occur once the patient received adjuvant therapy plus 10 g of AC per day? A study showed that autophagy of BC T47D cells was observed in vitro after treatment with ethanol extract of AC at a higher concentration (50 μg/mL) due to potent inhibition of CDK2 and CDK4.^[19]^ In addition, the inhibition of histone deacetylase activity was observed after treatment with ethanol extract of AC.^[19]^ Another study showed that 50 μg/mL ethanol extract of AC fruiting bodies containing 2 major compounds (dehydrosulfurenic acid and dehydroeburicoic acid) attenuated T47D BC cell activity by deregulating the PI3K/Akt/mTOR signaling pathway and key cell-cycle mediators (including CDK 4, CDK 6, cyclin B1, cyclin D3, retinoblastoma protein (Rb), and phosphorylated Rb), and inducing apoptosis.^[20]^ In fact, there are 2 major components, dehydrosulfurenic acid and dehydroeburicoic acid, in the AC supplied for the patient in this case report.^[21]^ Since PAL also acts as a CDK4 inhibitor, synergistic suppression might have induced autophagy when the patient received adjuvant therapy plus AC 10 g per day. This synergistic suppression may explain why PAL-FUL combined with AC showed the most potent influence on BC cells. In this case, unusual elevation of serum CA15-3 and LDH levels may be caused by the autophagy of BC cells due to the synergistic suppression of CDK4 in the cyclin D-CDK4/6-inhibitor of CDK4-Rb pathway. Massive quantities of damaged BC cells releasing CA15-3 and LDH shed into the circulation and subsequently result in the observation of elevated CA15-3 and LDH levels.^[11]^

## 4 Conclusion

In this case, the results of CTC analysis, LDH, ALP, and CA15-3 levels, and CT and MRI demonstrated that AC has anti-proliferative activity against BC, a synergistically cytostatic effect with adjuvant therapy, and alleviates myelo-suppressive effects. The observation of unusually continued elevation of CA15-3 and LDH levels but descending levels of ALP may indicate the potent efficacy of treatment resulting in massive tumor cell death. These effects may contribute to the improvement of quality of life and progression-free survival in patients with MBC. CTCs test results may be more sensitive than those of imaging tools used to monitor progression of BC and subsequently to evaluate the efficiency of therapy. Additionally, the safety of AC is also confirmed via the observations of the patient's normal liver and renal functions. Thus, AC could be a potent complementary medicine that promotes the therapeutic effects and improves outcomes of adjuvant therapy against BC with bone metastasis. The MBC patient is still alive with high quality of life so far, and we will follow up her status for at least the next 5 years. To our best knowledge, this is the first case report showing the enhancing and therapeutic effect of AC on adjuvant therapy in a patient with MBC.

## Acknowledgments

The authors thank Ming-Feng Li, Director of Abdominal Imaging, Department of Medical Imaging (Buddhist Tzu-Chi General Hospital, Hualien, Taiwan) for the comments regarding MRI and CT. We also thank Denny Roy, Senior Fellow and Supervisor of the POSCO Fellowship Program, Research Program, and Steven Michael Taub, Research Consultant (FBM UNIL, Lausanne, Switzerland) for their assistance with manuscript revision. We would like to thank Editage (www.editage.com) for English language editing. We thank Ms Shu-Chuan Lin and Ms Chiu-Mei Hsiang for their administrative assistance. We deeply appreciate the advanced BC patient (an intimate friend of the first author) for providing her medical records and data.

## Author contributions

**Conceptualization:** Ching-Feng Weng, Vesna Prijatelj.

**Data curation:** Vesna Prijatelj, Huei Long.

**Investigation:** Chi-Tan Hu, Huei Long.

**Methodology:** Vesna Prijatelj.

**Writing – original draft:** Huei Long, Vesna Prijatelj.

**Writing – review & editing:** Chi-Tan Hu, Ching-Feng Weng.
